# An Explainable Prediction for Dietary-Related Diseases via Language Models

**DOI:** 10.3390/nu16050686

**Published:** 2024-02-28

**Authors:** Insu Choi, Jihye Kim, Woo Chang Kim

**Affiliations:** 1 Department of Industrial and Systems Engineering, Korea Advanced Institute of Science and Technology, Daejeon 34141, Republic of Korea; jl.cheivly@kaist.ac.kr; 2 Department of Genetics and Biotechnology, College of Life Sciences, Kyung Hee University, Yongin 17104, Republic of Korea

**Keywords:** dietary pattern, obesity, dyslipidemia, language model, natural language processing (NLP), explainable artificial intelligence (xAI)

## Abstract

Our study harnesses the power of natural language processing (NLP) to explore the relationship between dietary patterns and metabolic health outcomes among Korean adults using data from the Seventh Korea National Health and Nutrition Examination Survey (KNHANES VII). Using Latent Dirichlet Allocation (LDA) analysis, we identified three distinct dietary patterns: “Traditional and Staple”, “Communal and Festive”, and “Westernized and Convenience-Oriented”. These patterns reflect the diversity of dietary preferences in Korea and reveal the cultural and social dimensions influencing eating habits and their potential implications for public health, particularly concerning obesity and metabolic disorders. Integrating NLP-based indices, including sentiment scores and the identified dietary patterns, into our predictive models significantly enhanced the accuracy of obesity and dyslipidemia predictions. This improvement was consistent across various machine learning techniques—XGBoost, LightGBM, and CatBoost—demonstrating the efficacy of NLP methodologies in refining disease prediction models. Our findings underscore the critical role of dietary patterns as indicators of metabolic diseases. The successful application of NLP techniques offers a novel approach to public health and nutritional epidemiology, providing a deeper understanding of the diet–disease nexus. This study contributes to the evolving field of personalized nutrition and emphasizes the potential of leveraging advanced computational tools to inform targeted nutritional interventions and public health strategies aimed at mitigating the prevalence of metabolic disorders in the Korean population.

## 1. Introduction

Disease prediction stands as a paramount research focus across myriad biomedical fields. Particularly, the prediction of food- and nutrition-related diseases assumes significant prominence in light of the rapid advancements in data mining and artificial intelligence (AI). Numerous studies have leveraged these advancements to explore disease prediction within the realm of food and nutrition. For instance, Shetty et al. (2017) applied Bayesian and K-Nearest Neighbor algorithms to diabetes patient databases, taking into account a multitude of diabetes attributes to predict the disease [1]. Similarly, Mir and Dhage (2018) constructed diabetes disease prediction classifier models using the Waikata Environment for Knowledge Analysis (WEKA), while Sisodia and Sisodia (2018) endeavored to devise a model capable of forecasting the likelihood of diabetes with maximum accuracy using Pima Indians Diabetes Database (PIDD) data [2,3]. Fitriyani et al. (2019) proposed a disease prediction model geared towards providing early predictions for type II diabetes and hypertension based on individual risk factor data [4]. Furthermore, Mishra et al. (2016) demonstrated that filter-based feature selection methods enhance the effectiveness of learning algorithms in diagnosing and predicting diabetes [5].

Building on this enduring interest in disease prediction, recent advancements have been made in the analysis of metabolic diseases. Particularly, dietary patterns have taken center stage in food and nutrition studies. Numerous investigations have underscored the profound impact of dietary patterns on metabolic diseases. For instance, Minich and Bland (2008) deliberated on various dietary strategies for managing metabolic syndrome, focusing on specific foods and nutritional phytochemicals known to influence insulin signaling [6]. Similarly, Kim et al. (2019) examined dietary patterns associated with obesity using the Korea National Health and Nutrition Examination Survey (KNHANES) data, while Ahluwalia et al. (2013) posited that dietary patterns offer a comprehensive, real-life approach to dissecting the intricate relationship between diet and disease [7,8].

Given this context, the current study seeks to predict representative metabolic diseases, such as obesity and dyslipidemia, by utilizing NLP techniques and AI algorithms from a dietary pattern perspective based on an individual’s one-day diet as single text and natural language processing (NLP) techniques. We also sought to verify the enhancement in predictive power provided by our NLP analysis using an explainable artificial intelligence (xAI) technique.

## 2. Method

### 2.1. Study Population

The KNHANES provides a critical foundation for analyzing dietary habits and health outcomes across various segments of the South Korean population. By categorizing participants across a spectrum of demographic variables from geographic location to socioeconomic status, this dataset enables a nuanced understanding of nutrition and health trends and disparities. Adhering to the ethical guidelines of the Declaration of Helsinki, the collection and use of KNHANES data underscore a commitment to ethical research practices, with the Korea Disease Control and Prevention Agency’s approval emphasizing the importance of these standards. Furthermore, written informed consent from all participants reinforces this ethical foundation.

In this study, we undertook a comprehensive data preprocessing phase of the 2016–2018 (KNHANES VII) dataset. The demographic information of KNHANES VII is presented in Table 1. The inclusion of data specific to 17 major cities and provinces facilitates in-depth analysis of regional dietary patterns and health outcomes, revealing the impact of local food availability, cultural preferences, and socioeconomic factors on dietary choices. This regional diversity is complemented by insights into urban versus rural living conditions, with distinctions between types of townships and housing conditions offering perspectives on how environmental factors influence nutritional habits and, by extension, health.

Demographic variables such as gender and age are integral to evaluating nutritional needs and health risks, while socioeconomic factors including income quantiles, education level, and occupational status provide a comprehensive framework for assessing how economic and social conditions affect dietary habits and health outcomes. The benchmark model developed from this demographic information focuses on forecasting health outcomes based on demographic factors alone. Compared to the benchmark model, our proposed model extends prior research [9,10] and highlights the continued relevance of KNHANES VII in Korean nutritional and health outcomes. 

### 2.2. Methodology

#### 2.2.1. Data Preprocessing

The first stage involved the elimination of erroneous entries, including instances where data were misplaced across columns and distortions in column values such as the listing of food items under the weight category. This action yielded 17,774 unique food items from the dataset.

Subsequent stages of normalization and cleaning were characterized by the removal of outliers—terms that were only represented once in the dataset. This was based on the presumption that such rarities would minimally impact the modeling process. Furthermore, efforts were made to standardize the food items by excluding English letters, numerals, and special symbols. This was performed to accommodate variations in the annotator’s entries. As variations in spelling may arise from factors such as accents or typographical errors, all food items were subjected to a Korean spelling test using Hanspell, a Python software version 1.0, based on NAVER’s Korean spelling checker, accessed on 19 February 2023 [11]. 

Post cleaning and standardization, the remaining dataset contained 1653 unique food items across 179,846 rows, which constituted 78.89 percent of the original dataset [12,13].

For the subsequent analysis, words were replaced with word vectors that encapsulated their meanings based on distributional semantics. The underlying hypothesis is that words in close proximity within a distribution share similar meanings. In this representation, each word is depicted as a continuous vector of a predetermined dimension in a variance table. 

In this study, we employed advanced word vectorization techniques to cluster similar food items, significantly streamlining the preprocessing phase and reducing the reliance on manual data handling. This method enhanced speed and accuracy compared with traditional manual data processing approaches. To enrich our analysis, we leveraged morpheme-processed corpora from Korean Wikipedia, Soynlp, and KorQuad, integrating these resources to refine our understanding of the linguistic structure of food names [14,15]. Recognizing the unique aspects of the Korean language, which can be segmented alphabetically, our analysis included a detailed consonant-level examination. Both consonant and syllable units were analyzed using advanced word embedding techniques, maintaining a 1:1 processing ratio.

The vector representation of food names in our study was visualized through a two-dimensional scatter plot, generated via Principal Component Analysis. This visualization, with the x- and y-axes representing reduced dimension vectors of food names, facilitated an intuitive understanding of the clustering of similar food items. Adjustments were implemented to address the diversity of food names found within specific clusters, utilizing the cluster numbers linked to each food name for reference. The examination of data from 2016 to 2018 led to the discovery of 835 unique food names. The incorporation of additional preprocessing methods, such as the Modu Corpus, and the exclusion of data from 2007–2009 (KNHANES IV), led to slight deviations in the final list of food names compared with those identified in the study by Choi et al. (2022) [9].

The final list of food names was derived by calculating the weighted average of each participant’s food intake frequency, which was then normalized by the frequency of keyword inclusion to yield a refined upper value of the computed value, ensuring a precise and accurate representation of food item relevance in the dataset. 

#### 2.2.2. Sentiment Analysis 

We leveraged sentiment analysis, a potent text mining technique, to probe the attitudes and opinions manifest in written or spoken content concerning food items. Sentiment analysis enables us to discern the tone of text—whether positive or negative—providing invaluable insights for examining a diverse range of content, from articles and movie reviews to social media posts [16,17].

To conduct sentiment analysis, we employed a machine learning strategy that integrates two sophisticated algorithms: the Convolutional Neural Network (CNN) [18] and the Bidirectional Long-Short Term Memory (BiLSTM) network [19]. The synergy of CNN and BiLSTM in our combined CNN-BiLSTM model facilitates the computation of a sentiment score for textual content related to food, shedding light on the positive or negative sentiments tied to specific food names. This sentiment score is quantified using a mathematical function that assigns “1” to positive sentiments and “0” to negative sentiments.

The CNN algorithm, typically associated with image classification tasks, is repurposed in our study as a text analysis tool, enabling the detection of patterns within text data. Conversely, the BiLSTM model excels in processing sequential data, making it perfectly suited for text analysis where the sequence of words plays a pivotal role. This integration leverages the strengths of both models in sequence processing and pattern recognition, significantly enhancing the accuracy of sentiment analysis in our research.

In the architecture of our CNN-BiLSTM model, various layers are meticulously designed to process and analyze text data effectively. Starting from an input layer that receives the text, the process progresses through an embedding layer that converts words into meaningful numerical vectors. The architecture is further strengthened with dropout layers to prevent overfitting and dense layers that facilitate decision-making, culminating in a model with 806,189 trainable parameters. This reflects the model’s complexity and its capacity to learn intricately from text data.

For the model’s initialization, we opted for the He initialization method, renowned for its efficacy in models employing ReLU activation functions [20]. We selected the AdamW optimizer, an enhancement of the classic Adam optimizer, for its refined ability to adjust learning rates and decouple weight decay from gradient updates, thus optimizing model training efficiency [21].

We conceptualized entire menus as discrete textual entities, synthesizing sentiment analysis into a singular metric—hereafter referred to as the “sentiment score”. This innovative approach allowed us to interpret concatenated menu items as unified textual segments, thereby streamlining the extraction and analytical process of sentiment values. This method provides a refined means of examining the relationship between dietary choices and health conditions, such as diabetes and dyslipidemia, by distilling complex data into a single, interpretable dimension.

#### 2.2.3. Dietary Pattern Extraction

We utilized Latent Dirichlet Allocation (LDA) [22], a robust tool in the domain of natural language processing (NLP), to analyze textual data from the KNHANES VII conducted between 2016 and 2018. LDA is particularly effective at revealing hidden patterns, themes, or structures within extensive volumes of text, making it exceptionally suited for analyzing the complex and diverse dietary information gathered in the survey.

LDA operates under the assumption that documents (for our purposes, individual dietary records) consist of a blend of topics (which we will refer to as “patterns”), with a pattern being identified as a group of words that commonly appear in conjunction. In the analysis of text data, LDA assigns each word in a document to a specific pattern based on the probability of the word belonging to that pattern. This assignment is guided by two principal parameters: one that determines the likely number of patterns to be found in each document and another that dictates the number of words expected in each pattern. These parameters are crucial for fine-tuning the model to mirror the data’s inherent structure accurately.

The application of LDA in our study facilitated a systematic reduction in high-dimensional dietary information into a concise set of patterns that accurately reflect the population’s dietary habits. Being an unsupervised machine learning model, LDA does not necessitate predefined categories, thereby allowing for the unbiased identification of natural groupings of dietary information based on the actual data.

Our implementation of LDA employed Gibbs sampling, a statistical method that iteratively assigns words to patterns until a stable distribution of patterns across documents is achieved. This method is particularly beneficial for accurately capturing the subtle nuances of dietary patterns within the population.

The primary aim of applying LDA to the dietary data was to condense the extensive and varied information into distinct dietary patterns that could be analyzed in relation to health outcomes such as obesity and dyslipidemia. By identifying these patterns, we strive to enhance the understanding of the association between various dietary habits and these health conditions. 

After identifying dietary patterns, we determined the optimal number of dietary patterns to ensure that the patterns identified were both statistically robust and meaningful within the context of dietary research. To select the optimal number of patterns for the LDA model, we evaluated both perplexity and topic coherence metrics [23,24]. In our study, we implemented a unique approach to analyze dietary patterns by utilizing binary columns corresponding to the identified patterns. For each individual, if their diet aligns with a specific pattern, the column representing that pattern is marked with a “1”, while all other pattern columns are set to “0”. This method ensures that for each row—representing an individual’s dietary data—only one column receives a “1”, indicating the predominant dietary pattern for that person. This binary system allowed us to clearly and efficiently categorize dietary habits into distinct patterns, facilitating a focused analysis of how each pattern is related to health outcomes like obesity and dyslipidemia.

#### 2.2.4. Target Diseases’ Definitions

In our analytical framework, the classification targets—obesity and dyslipidemia—are defined using precise criteria reflective of the health challenges pertinent to the Asia-Pacific demographic standards. 

Obesity is classified when an individual’s Body Mass Index (BMI) reaches or exceeds a threshold of 25. This benchmark is in accordance with the criteria established for populations in the Asia-Pacific region [25].

Dyslipidemia is identified through a comprehensive evaluation of lipid profiles, marked by one or more of the following conditions [26]:

- A triglyceride (TG) level at or exceeding 200 mg/dL, or a total cholesterol level surpassing 240 mg/dL, indicating elevated lipid concentrations that pose significant health risks.
- An HDL-cholesterol (high-density lipoprotein cholesterol) level falling below the threshold of 40 mg/dL in males or 50 mg/dL in females, reflecting the protective lipid’s insufficiency against cardiovascular diseases.
- An LDL-cholesterol (low-density lipoprotein cholesterol) level at or above 160 mg/dL, highlighting an increased risk of atherosclerotic cardiovascular events. If TG levels were below 400 mg/dL, LDL-cholesterol was calculated using the Friedewald formula to recalibrate the LDL-cholesterol value [27].

#### 2.2.5. Machine Learning-Based Classification

We used a machine learning approach to predict obesity and dyslipidemia by analyzing dietary data. This comprehensive methodology integrates advanced machine learning algorithms for classification, sentiment analysis for nuanced data interpretation, and statistical testing to validate the performance of our models. Below is a logical reorganization and expansion of our research methodology, incorporating the evaluation of model performance and the application of statistical tests.

Our analysis began with the application of balanced accuracy (BA) and the F1 score as the primary metrics to evaluate model performance, addressing the challenge of imbalanced data representation of obesity (32.9%) and dyslipidemia (45.7%). These metrics were derived from the general confusion matrix using the following formulas:

(1) $$Balanced\;Accuracy=\frac{\left(\frac{True\;Positive}{True\;Positive+False\;Negative}+\frac{True\;Negative}{True\;Negative+False\;Positive}\right)}{2}$$

(2) $$F1\;Score=\frac{2\times True\;Positive}{2\times True\;Positive+False\;Positive+False\;Negative}$$

The performance measures represent the average outcomes of ten cross-fold tests, accompanied by 95% confidence intervals, ensuring the robustness and reliability of our findings.

To classify obesity and dyslipidemia, we strategically employed three gradient-boosting algorithms: XGBoost [28], LightGBM [29], and CatBoost [30]. Each was chosen for its specific strengths in handling the complexities of dietary data, ensuring a comprehensive analysis. XGBoost is valued for its efficiency with large datasets, LightGBM for its speed and precision across a wide range of dietary variables, and CatBoost for its adept handling of categorical data, which is prevalent in dietary studies. The combined use of these models enhances the robustness of our experiments, providing a multifaceted approach to ensure reliable and generalizable findings in assessing the relationship between diet and these health conditions. We trained 50 epochs for each set with the early stopping method and randomly divided the whole dataset into ten sets. We used eight sets as the training set, one set as the validation set, and the remaining one set as the test set. For the nine sets, we used k-fold cross-validation. For the performance evaluation, we used binary cross-entropy.

We digitized the data by converting textual food item names into a format compatible with deep learning and machine learning methodologies. This digitization process involved vectorizing the dataset’s vocabulary, primarily composed of food names, into high-dimensional numerical vectors through one-hot encoding. This technique ensures that each unique food item is distinctly represented, enabling further analysis.

To enhance the specificity of our analysis, we employed the Term Frequency-Inverse Document Frequency (TF-IDF) technique for vectorization. The TF-IDF method effectively accentuates the importance of various food items within the dietary logs, weighing them by both their frequency and their uniqueness across all entries. This process yields a nuanced numerical representation that captures the significance of each food item within the broader context of dietary patterns.

To strengthen the robustness and applicability of our findings, we implemented a 10-fold cross-validation scheme. By partitioning the data into ten subsets and iteratively using each subset for validation and the rest for training, we mitigated the risk of overfitting and enhanced the stability of our model’s performance metrics. Our focus on cross-validation ensured that our model evaluations—reflected in balanced accuracy and F1 scores—were both stable and reliable.

The optimization of our models was carried out using the Optuna [31] library, conducting a series of ten trials with balanced accuracy set as the optimization target. This meticulous tuning was instrumental in refining the predictive capabilities of our models. For interpretability, a critical component for actionable insights, we integrated Tree-SHAP [32,33], which enabled us to measure how individual dietary factors influenced the health outcomes being investigated.

In addition to the models’ performance, sentiment analysis was incorporated to classify the dietary records, enriching the dataset with insights derived from natural language processing. This sentiment analysis was trained across 50 epochs for each data subset, utilizing early stopping to prevent overfitting. The dataset was randomly divided into ten sets—eight for training, one for validation, and one for testing—with k-fold cross-validation applied to the remaining nine sets. Performance evaluation was conducted using binary cross-entropy, providing a measure of the model’s ability to distinguish between the binary classification targets.

To validate the effectiveness of incorporating NLP-based indices into our models in improving the prediction of obesity and dyslipidemia, we conducted a paired t-test comparing the performance of the benchmark model against the model enhanced with the NLP indices. Statistical analysis was performed using the Python package “scipy,” version 1.0, accessed on 19 February 2023 [34]. We indicated “***” for *p*-values of the paired *t*-test below 0.01, “**” for *p*-values of 0.01 or higher but below 0.05, and “*” for *p*-values of 0.05 or higher but below 0.1 and did not mark anything otherwise.

## 3. Results

The study participants’ demographic summaries are in Table 2. Our study encompasses a diverse cohort of 16,809 participants drawn from KNHANES VII dataset. The demographic breakdown reveals a slight female majority, with 57.5% female participants and 42.5% male. The age distribution is fairly even across the adult lifespan, with 32.7% aged between 19 and 39 years, 35.9% between 40 and 59 years, and the remaining 31.4% aged 60 years and above.

In terms of living arrangements, a slight majority of 53.4% reside in apartments, with the remainder 46.6% living in general housing. Educational attainment among the participants varies, with 19.6% having graduated from elementary school, 37.8% from high school, and 33.5% holding an associate degree or higher.

Within the participant population, health indicators show that obesity is present in 32.9% of individuals, and dyslipidemia is observed in 45.7%. These figures indicate that more than a third of the surveyed group is affected by these metabolic disorders, underscoring the significant impact on public health.

### 3.1. Dietary Pattern Extraction Results

In our comprehensive analysis utilizing LDA, we unearthed three prominent dietary patterns from the data collected from the Korean participants. These patterns offer a panoramic view of the prevailing food choices and their implications for health and nutrition.

**Figure 1:**
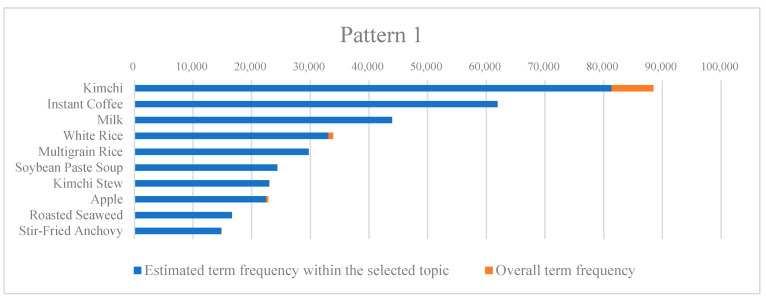

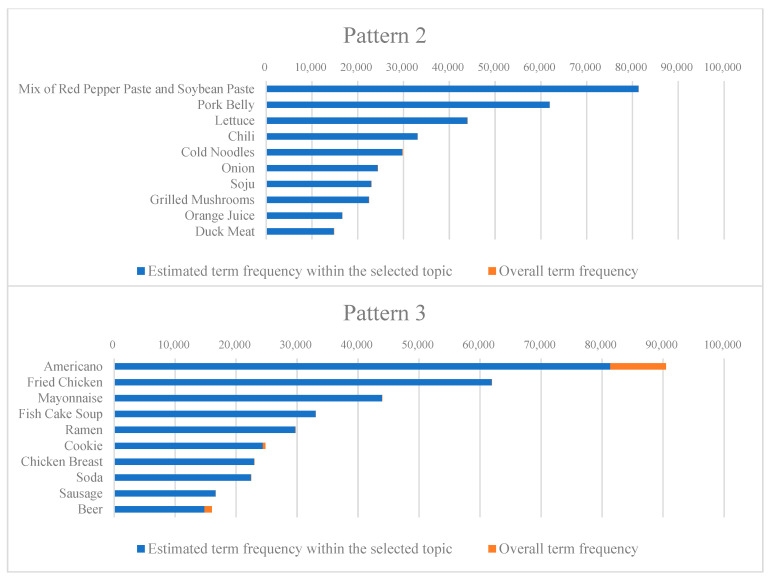
Estimated term frequency within patterns and overall term frequency of the top 10 foods for each pattern.

Table 3 delineates the most frequently occurring food items within each identified pattern, and Figure 1 depicts the estimated term frequencies for the top ten food characteristics of each pattern. The first pattern, which can be described as “Traditional and Staple”, is replete with items such as “Kimchi”, “Instant Coffee”, “Milk”, “White Rice”, and “Multigrain Rice”. This pattern is emblematic of a diet steeped in Korean culinary heritage, with a staggering 67.1% of participants and 62.3% of the textual mentions aligning with this group. The prevalence of this pattern underscores the quintessential role these foods play in daily consumption and reflects the cultural gastronomic identity. The dietary preferences exhibited here also shed light on the potential health benefits and drawbacks associated with a diet rich in rice-based dishes and fermented products.

The second pattern, labeled as “Communal and Festive”, captures the essence of Korean social and celebratory dining experiences, spotlighting items like “Mix of Red Pepper Paste and Soybean Paste”, “Pork Belly”, “Lettuce”, and “Cold Noodles (Naengmyeon)”. Accounting for 17.4% of participants, this pattern likely mirrors the convivial nature of Korean meals, where savory meats and piquant side dishes are central to social gatherings and festivities. This pattern is particularly insightful for understanding the nutritional nuances of communal eating habits, including the preference for richer, more flavorful dishes.

The third pattern, termed “Westernized and Convenience-Oriented”, reflects a shift towards globalized food preferences, with items such as “Americano”, “Fried Chicken”, “Mayonnaise”, and “Ramen” taking center stage. This pattern, embraced by 15.5% of participants, is indicative of an inclination towards fast food and processed products. The significant representation of these items, coupled with a notable 22.7% of textual mentions, raises critical considerations regarding their influence on public health issues, including obesity and metabolic disorders.

### 3.2. Predicting Diseases and Analyzing Disease Prediction Results

#### 3.2.1. Obesity Prediction Results

In our rigorous examination of obesity prediction methodologies, we scrutinized the efficacy of three state-of-the-art machine learning algorithms—XGBoost, LightGBM, and CatBoost. As detailed in Table 4, our empirical findings reveal a marked elevation in predictive accuracy following the integration of NLP-based indices, particularly sentiment scores and pattern-based binary variables.

When juxtaposed against the baseline benchmark model, our enriched models, infused with these NLP-derived indices, exhibited substantial gains in the key performance indicators. Remarkably, the assimilation of sentiment scores and pattern-based binary variables bolstered model efficacy. Illustratively, within the XGBoost framework, we observed an increase in balanced accuracy from 0.5276 to 0.5879 and an augmentation of the F1 score from 0.4958 to 0.5813.

The statistical significance of these enhancements was corroborated by paired *t*-tests, yielding T-statistics that spanned from 13.6947 to 18.7120 and *p*-values at a trifling 0.0000 ***, decisively refuting all null hypotheses at the stringent alpha threshold of 0.01. This unambiguously signifies that incorporating NLP-based indices fortifies the prognostic capabilities of our models in discerning obesity risk.

**Figure 2:**
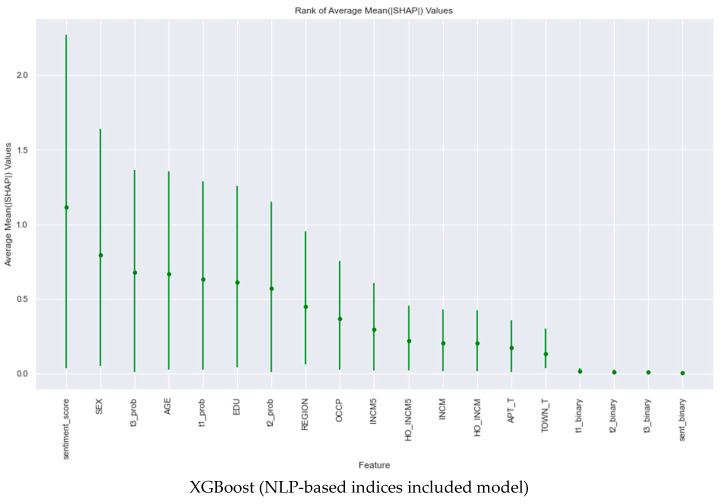

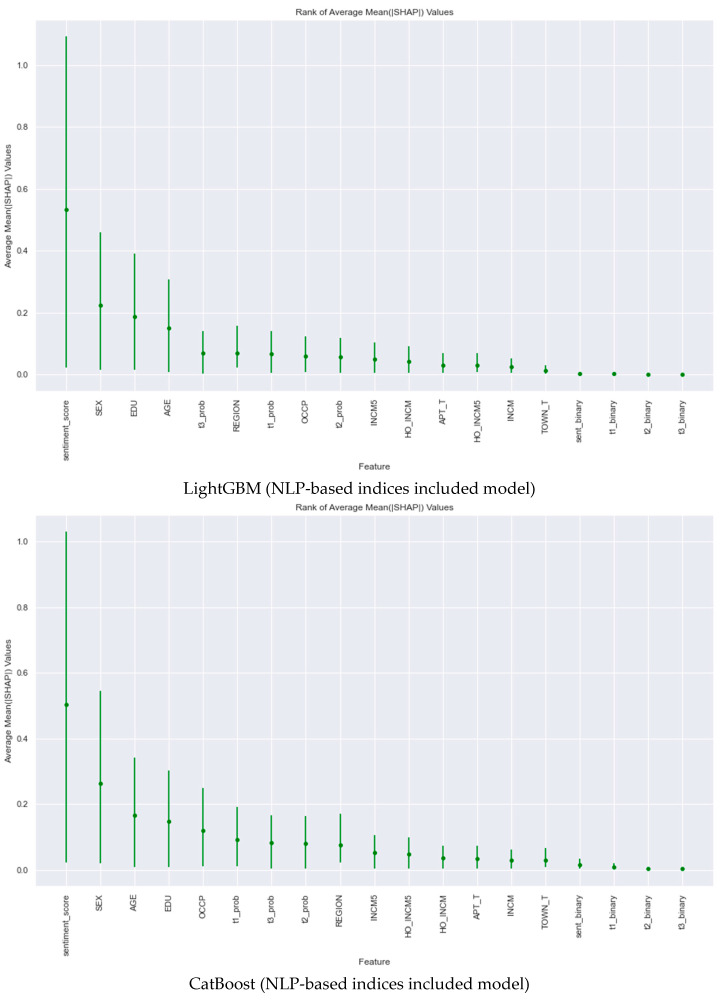
Mean absolute SHAP values of the ten models used to predict obesity.

Although not visually presented here, Figure 2 is posited to articulate the mean absolute SHAP values across the models, shedding light on the contribution of individual features to the predictive acumen. Applying SHAP values, with a 95% confidence interval and predicated on nine degrees of freedom, affords nutrition professionals a simple and interpretable understanding of the determinants influencing the model outcomes. Our analysis confirmed that the influence of “sentiment_score” was consistently profound across all models in predicting obesity. Additionally, while the impact of pattern-based binary variables was relatively modest, their positive values nonetheless contributed to the predictive process, thereby validating the significance of our NLP-based variables in forecasting obesity.

#### 3.2.2. Dyslipidemia Prediction Results

Table 5 presents the comparative results for dyslipidemia prediction using the XGBoost, LightGBM, and CatBoost algorithms, considering both the benchmark models and those incorporating NLP-based indices. Notably, the introduction of NLP-based indices—such as sentiment scores and pattern-based binary variables—yielded improvements in the balanced accuracy and F1 score across the models. For instance, the XGBoost model exhibited an increase in balanced accuracy from 0.5497 to 0.5956 and an increase in the F1 score from 0.5461 to 0.5937.

These enhancements were statistically significant, with *p*-values indicating a decisive rejection of the null hypotheses, particularly at an alpha level of 0.1. However, the magnitude of improvement was less pronounced in dyslipidemia than in obesity, with some measures showing no significant difference depending on the alpha level chosen.

**Figure 3:**
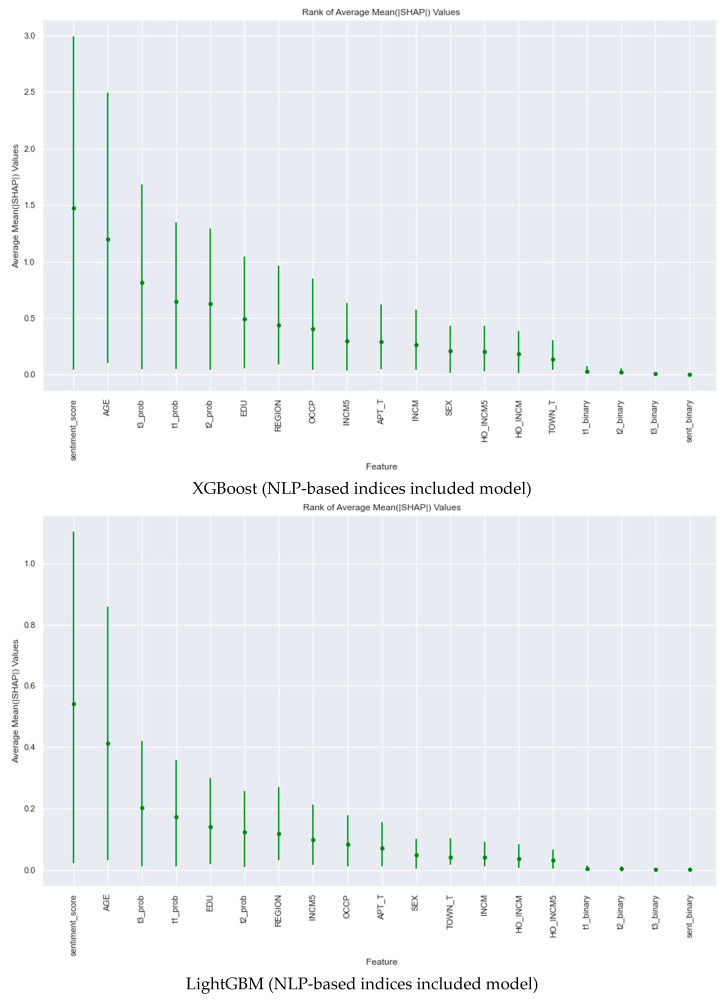

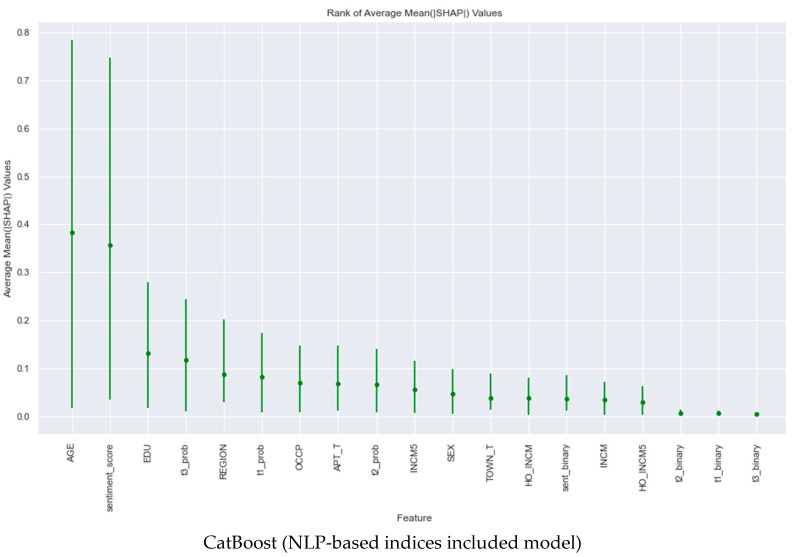
Mean absolute SHAP values of the ten models used to predict dyslipidemia.

The experimental findings further highlighted the substantial impact of the sentiment score in predicting instances of dyslipidemia. In contrast, age emerged as the most influential feature in the CatBoost model for dyslipidemia prediction. These insights are visually corroborated in Figure 3, which illustrates the mean absolute SHAP values across the ten models. 

The influence of dietary patterns on health outcomes was also underscored, with Pattern 3—characterized by a high frequency of fast foods and convenience items—showing a particularly strong association with both obesity and dyslipidemia. This pattern’s significant predictive value is consistent with the existing literature linking such dietary choices to metabolic health issues.

The probability of adhering to a rice-based diet (Pattern 1) and a meat-based diet (Pattern 2) was also found to be a significant determinant in the models’ predictions. This finding suggests that specific dietary patterns may play a crucial role in the onset and progression of metabolic diseases.

## 4. Discussion

Our study applied NLP techniques to discern three distinct dietary patterns among Korean adults, offering a novel lens through which to view the intricate relationship between diet and metabolic health outcomes utilizing data from KNHANES VII. In our study, LDA analysis revealed three distinct dietary patterns among Korean participants, providing a comprehensive overview of dietary preferences and their health implications. The “Traditional and Staple” pattern, predominant among 67.1% of the participants, features staples like Kimchi and rice, highlighting the cultural significance of these foods in daily Korean diets and their potential health impacts. The “Communal and Festive” pattern, identified in 17.4% of the participants, emphasizes foods associated with social gatherings, such as Pork Belly and Cold Noodles, reflecting the social aspect of eating and its nutritional implications. Lastly, the “Westernized and Convenience-Oriented” pattern, chosen by 15.5% of the participants, comprises fast food and processed items, indicating a trend towards Westernized dietary preferences and their potential risks for public health, particularly obesity and metabolic disorders. These findings underscore the varied dietary landscapes in Korea and their complex relationships with health outcomes, offering valuable insights for targeted nutritional interventions and public health strategies.

Moreover, our findings demonstrate a significant improvement in the predictive models for obesity and dyslipidemia when incorporating NLP-based indices, including sentiment scores and dietary patterns, compared with traditional benchmark models. This advancement was observed across all three employed machine learning techniques (XGBoost, LightGBM, and CatBoost), highlighting the robustness of NLP methodologies in enhancing disease prediction accuracy.

This research underscores the importance of considering dietary patterns, identified through sophisticated NLP techniques, as key indicators for metabolic diseases. By integrating these dietary patterns and sentiment scores into our predictive models, we achieved statistically significant improvements in forecasting obesity, a testament to the potential of leveraging NLP-based insights in public health and nutritional epidemiology. 

Consequently, our study forges a pioneering integration of NLP techniques with conventional epidemiological methodologies, aiming to unravel the impact of dietary intake on metabolic disorders. Utilizing data from KNHANES VII, spanning the years 2016 to 2018, we applied sentiment analysis and LDA to deconstruct and interpret the intricate narrative of dietary habits. This approach facilitated the extraction of sentiment scores and the identification of diet-related patterns, each quantified with their corresponding probabilities.

The dietary habits of Korean adults observed in our study resonate with behaviors documented in preceding research [35,36]. Our predictive methodology for obesity and dyslipidemia, grounded in food data, corroborates with prior findings. Notably, a “White Rice and Kimchi pattern”, which aligns with Pattern 1, a rice-based traditional dietary pattern, is associated with obesity in Korean adults and dyslipidemia, including manifestations such as dyslipidemia and reduced high-density lipoprotein cholesterol levels [36]. Conversely, a dietary pattern distinguished by a high intake of whole grains, legumes, fruits, and seaweed was inversely related to obesity [37]. Similarly, dietary patterns characterized by high consumption of meat and alcohol, akin to Patterns 2 and 3, were found to influence dyslipidemia adversely. In contrast, a diet rich in grains, vegetables, and fish was associated with a reduced risk of developing dyslipidemia and metabolic syndrome [38]. 

Our study contributes to the field of nutritional science by exploring the application of AI and NLP techniques. This approach has shown promising potential in supporting the management of metabolic disorders, including obesity and dyslipidemia. The methodological progress we have made encourages further exploration into the integration of artificial intelligence within nutritional science, offering insights that could be valuable for the evolving practice of personalized nutrition.

Applying sentiment analysis and topic modeling to dietary intake data in our study has provided a glimpse into the capacity of NLP methodologies to aid in predicting obesity and dyslipidemia among the Korean population. The outcomes of this research suggest that computational tools may play a supportive role in advancing nutritional epidemiology and public health, enriching our understanding and approach to these complex health issues.

The innovation in our data preprocessing for food menus marks a significant advancement in nutritional research. By streamlining this process, we not only reduced the workload and time required to prepare and analyze dietary data but also significantly increased the speed at which new information can be incorporated into our analysis [35,39]. This rapid assimilation of data is crucial in the fast-paced field of nutrition, where dietary trends and health recommendations evolve continuously. Our method ensures that the insights we derive are both relevant and reflective of the latest dietary behaviors and preferences.

This enhanced processing capability is particularly beneficial for personalized nutrition, a field that relies heavily on up-to-date, individualized data to offer bespoke dietary advice. By efficiently processing large volumes of dietary information, our approach supports the creation of dynamic, personalized nutrition plans that can quickly adapt to changes in an individual’s dietary habits, health status, or emerging scientific evidence. It enables a more agile response to the unique nutritional needs and preferences of individuals, facilitating a more targeted and effective approach to dietary counseling and intervention.

Furthermore, our preprocessing method opens the door to more comprehensive analyses of dietary patterns and their health implications. By efficiently handling diverse and complex dietary data [36,40,41], we can explore deeper relationships between food intake and health outcomes across different populations. This capability allows for a broader understanding of how specific dietary components contribute to or mitigate the risk of health conditions such as obesity and dyslipidemia, providing a solid foundation for developing more effective public health guidelines and nutritional therapies.

In addition, the ability to quickly process and analyze new dietary surveys means that our nutritional recommendations can stay at the forefront of scientific discovery and public health trends. This not only enhances the accuracy and relevance of our advice but also ensures that it is grounded in the most current evidence available. As a result, individuals can make informed decisions about their diet and lifestyle, empowered by the knowledge that their choices are backed by the latest in nutritional science.

In essence, the streamlined data preprocessing method we have developed represents a leap forward in the ability to deliver precise, personalized nutrition advice. By enabling the rapid integration and analysis of dietary data, we are paving the way for a more responsive, evidence-based approach to nutrition that can significantly impact individual and public health outcomes.

Our deployment of NLP, mainly through the LDA algorithm, which is used in various areas [42], marks a significant stride in understanding the nuanced architecture of dietary habits beyond the conventional scope identified in related previous works on dietary patterns [43,44,45,46,47]. This advanced analysis permits a dive into the complex mosaic of food intake and preferences without the constraints of pre-established dietary categorizations. It is especially pivotal for the burgeoning field of personalized nutrition, which thrives on the identification and understanding of distinctive dietary patterns unique to individuals.

This methodological innovation transcends traditional dietary analysis by unlocking a more dynamic and detailed exploration of dietary habits. By analyzing textual data from dietary records, the LDA algorithm facilitates the discovery of underlying dietary themes or patterns that might not be immediately apparent. This capability is indispensable in personalized nutrition, where the goal is to tailor dietary advice to the specific health requirements, lifestyle, and even genetic predispositions of individuals. The precise identification of dietary behaviors through our NLP approach enables a more nuanced development of dietary recommendations, which can be finely tuned to align with individual health goals and nutritional needs.

Furthermore, our NLP-driven analysis enriches the dialogue on dietary interventions by providing a basis for more informed decision-making in nutritional counseling. The insights gleaned from the LDA analysis offer a detailed picture of an individual’s dietary landscape, highlighting potential areas for nutritional optimization. For instance, the identification of a prevalent reliance on processed foods or a deficiency in fruit and vegetable intake can inform targeted dietary interventions aimed at mitigating disease risk or addressing specific health concerns.

Moreover, the application of NLP and LDA in our study augments the precision of personalized nutrition plans and contributes to the broader discourse on dietary health and chronic disease prevention. By elucidating the intricate relationships between various dietary patterns and health outcomes, our methodology paves the way for more effective public health strategies and nutritional education programs. It champions the cause of precision nutrition by advocating for dietary advice that is not only scientifically sound but also personally relevant and actionable.

In essence, our approach embodies a significant advancement in nutritional research methodology, leveraging the power of AI to delve deeper into the complex web of human dietary habits. It stands as a testament to the potential of integrating technology and nutritional science to foster a deeper understanding of diet and health, marking a pivotal step towards the realization of truly personalized nutrition.

Our research took a novel approach by targeting obesity and dyslipidemia, assigning a binary value (1 or 0) to each condition based on the presence or absence of these metabolic disorders. Through the application of sentiment analysis, we ventured beyond traditional analysis to understand how the sentiment scores, approaching closer to 1, correlate with an increased likelihood of developing obesity or dyslipidemia. This methodology underscores a crucial aspect of our study, highlighting the capability of sentiment analysis not merely as a tool for gauging general attitudes but as a predictive instrument that can signal potential health risks associated with dietary patterns.

By focusing on sentiment scores derived from dietary data, we were able to uncover a significant relationship between the positivity of dietary sentiments and the increased risk of obesity and dyslipidemia. This finding is instrumental in the realm of personalized nutrition, where it becomes clear that understanding the nuanced sentiments individuals hold towards certain foods or dietary habits can offer predictive insights into their health outcomes. For instance, a higher sentiment score towards foods high in saturated fats and sugars may indicate a predisposition to obesity and dyslipidemia, providing a valuable indicator for nutrition professionals to identify at-risk individuals.

In summing up our discussion, by integrating the cutting-edge methodologies of artificial intelligence, specifically natural language processing and sentiment analysis, with the nuanced domain of nutritional science, our study endeavors to contribute meaningfully to the dynamic field of personalized nutrition. We strive to highlight their strengths by applying their insights to predict dietary trends.

Our exploration into the predictive potential of sentiment analysis, particularly in relation to obesity and dyslipidemia, offers a fresh perspective on the interplay between dietary habits and metabolic health. This novel application of AI techniques in nutritional research aims not only to enrich our understanding of diet-related health outcomes but also to enhance the precision and personalization of dietary interventions. It is our hope that these methodological advancements will encourage further inquiry and innovation at the intersection of technology and nutrition, fostering a deeper, more holistic understanding of the factors that influence dietary choices and their health consequences [48,49,50].

## 5. Conclusions

We embarked on an interdisciplinary journey that melded the realms of artificial intelligence, specifically NLP techniques, with the intricate field of nutritional science. Our objective was to illuminate the pathways through which dietary patterns influence the prevalence of obesity and dyslipidemia within the South Korean population. By harnessing the vast and detailed dataset provided by the KNHANES VII, we sought to unravel the complex relationship between diet and these metabolic disorders.

Our approach was twofold: firstly, we applied sentiment analysis to parse the dietary data, assigning sentiment scores reflecting the positive or negative connotations associated with food items. This novel application of sentiment analysis gave us a unique lens to examine how emotional and perceptual attitudes towards certain foods could influence health outcomes. Secondly, through the use of LDA, we identified distinct dietary patterns within the population and used them as the features for forecasting obesity and dyslipidemia, offering a nuanced understanding of the prevalent eating habits and their potential health implications.

The integration of these advanced analytical techniques allowed us to generate predictive models that not only offer insights into the dietary determinants of obesity and dyslipidemia but also enhance the precision of disease forecasting. Our findings underscore the significant role that dietary patterns play in the development of metabolic disorders, reinforcing the notion that a deeper understanding of these patterns is crucial for effective public health interventions and personalized nutrition.

Our study highlights the transformative potential of combining AI methodologies with nutritional epidemiology. By employing NLP and sentiment analysis, we were able to dissect the complex web of dietary information, revealing the intricate ways in which food consumption is intertwined with health outcomes. This methodology offers a promising avenue for future research to elucidate the connections between diet and disease, paving the way for more targeted and effective nutritional guidance.

In conclusion, our study represents a step forward in the application of AI in nutritional science, offering novel insights into the dietary determinants of obesity and dyslipidemia. It underscores the potential of interdisciplinary approaches to enhance our understanding of the complex relationship between diet and health, advocating for integrating technological innovations into nutritional research and practice.

## Figures and Tables

**Table 1:** Demographic information of KNHANES VII data.

Column	Description	Category
REGION	17 cities	Seoul; Busan; Daegu; Incheon; Gwangju; Daejeon; Ulsan; Sejong; Gyeonggi; Gangwon; Chungbuk; Chungnam; Jeonbuk; Jeonnam; Gyeongbuk; Gyeongnam; Jeju.
TOWN_T	Townships	Dong; Eup/Myeon.
APT_T	Apartment	Non-apartment; Apartment.
SEX	-	Male; Female.
AGE		1–80 *
INCM	Income quantiles (individual)	Low; Medium low; Medium high; High.
HO_INCM	Income quantiles (household)	Low; Medium low; Medium high; High.
INCM5	Income quintiles (individual)	Low; Medium low; Medium; Medium high; High.
HO_INCM5	Income quintiles (household)	Low; Medium low. Medium; Medium high; High.
EDU	Education level	Graduated elementary school or lower; Graduated middle school-; Graduated high school; Graduated college or higher.
OCCP	Occupational reclassification and unemployment/non-economic activities (except conscripted soldiers)	Managers, professionals, and related personnel; Office workers; Service and sales workers; Skilled workers in agriculture, forestry, and fisheries; Skill personnel and equipment and machine operation and assembly workers; Simple labor workers; Unemployed (housewife, student, etc.).

* 80 years of age or older marked as 80.

**Table 2:** Summary of study participants.

Feature	Category	Feature	Proportion (%)
Sex	Demographic	Male	7144 (42.5%)
		Female	9665 (57.5%)
Age	Demographic	19–39	5296 (32.7%)
		40–59	5814 (35.9%)
		60+	5077 (31.4%)
House type	Demographic	General	7831 (46.6%)
		Apartment	8978 (53.4%)
Highest level of education	Demographic	Graduated elementary school	3288 (19.6%)
		Graduated high school	6342 (37.8%)
		Over associate degree/bachelor’s degree	5639 (33.5%)
Obesity	Disease	Obesity	5535 (32.9%)
		Normal	11,311 (67.1%)
Dyslipidemia	Disease	Dyslipidemia	1350 (45.7%)
		Normal	1605 (54.3%)
Total		16,809	

**Table 3:** Most frequent keywords that appeared in the patterns.

Rank	Pattern 1	Pattern 2	Pattern 3
1	Kimchi	Mix of Red Pepper Paste and Soybean Paste	Americano
2	Instant Coffee	Pork Belly	Fried Chicken
3	Milk	Lettuce	Mayonnaise
4	White Rice	Red Pepper	Fish Cake Soup
5	Multigrain Rice	Cold Noodle	Ramen
6	Soybean Paste Soup	Onion	Salty Snack (Cookie)
7	Kimchi Stew	Soju	Chicken Breast
8	Apple	Grilled Mushrooms	Soda
9	Roast Seaweed	Orange Juice	Sausage
10	Stir-Fried Anchovy	Duck Meat	Beer
Proportion of participants	67.1%	17.4%	15.5%
Proportion of tokens	62.3%	15.0%	22.7%

**Table 4:** Performance comparison results of obesity prediction results.

Machine Learning Model	Performance Measure	Benchmark Model	NLP-Based Indices Included Model	T-Statistic	*p*-Value
XGBoost	Balanced accuracy	0.5276	0.5879	15.2015	0.0000 ***
	F1 score	0.4958	0.5813	13.6947	0.0000 ***
LightGBM	Balanced accuracy	0.5194	0.5855	15.1472	0.0000 ***
	F1 score	0.4752	0.5754	18.7120	0.0000 ***
CatBoost	Balanced accuracy	0.5276	0.5879	15.2015	0.0000 ***
	F1 score	0.4958	0.5813	13.6947	0.0000 ***

Note: We indicated “***” for *p*-values of the paired t-test below 0.01.

**Table 5:** Performance comparison results of dyslipidemia prediction results.

Machine Learning Model	Performance Measure	Benchmark Model	NLP-Based Indices Included Model	T-Statistic	*p*-Value
XGBoost	Balanced accuracy	0.5497	0.5956	3.8721	0.0019 ***
	F1 score	0.5461	0.5937	4.0133	0.0015 ***
LightGBM	Balanced accuracy	0.5730	0.5873	1.6280	0.0690 *
	F1 score	0.5676	0.5858	2.2078	0.0273 **
CatBoost	Balanced accuracy	0.5801	0.6186	3.5572	0.0031 ***
	F1 score	0.5741	0.6166	3.8686	0.0019 ***

Note: We indicated “***” for *p*-values of the paired t-test below 0.01, “**” for *p*-values of 0.01 or higher but below 0.05, and “*” for *p*-values of 0.05 or higher but below 0.1 and did not mark anything otherwise.

## Data Availability

Publicly available datasets were analyzed in this study. This data can be found here: https://knhanes.kdca.go.kr/knhanes/sub03/sub03_02_05.do. In this study, we undertook a comprehensive data preprocessing phase of the 2016–2018 (KNHANES VII) dataset.
